# Chronic diazepam administration increases the expression of Lcn2 in the CNS

**DOI:** 10.1002/prp2.283

**Published:** 2017-01-31

**Authors:** Tomonori Furukawa, Shuji Shimoyama, Yasuo Miki, Yoshikazu Nikaido, Kohei Koga, Kazuhiko Nakamura, Koichi Wakabayashi, Shinya Ueno

**Affiliations:** ^1^ Department of NeurophysiologyHirosaki University Graduate School of MedicineHirosakiJapan; ^2^ Research Center for Child Mental DevelopmentHirosaki University Graduate School of MedicineHirosakiJapan; ^3^ Department of NeuropathologyHirosaki University Graduate School of MedicineHirosakiJapan; ^4^ Department of NeuropsychiatryHirosaki University Graduate School of MedicineHirosakiJapan

**Keywords:** Benzodiazepine, diazepam, GABA_A_‐Rs, lcn2, Ngal, transcriptome

## Abstract

Benzodiazepines (BZDs), which bind with high affinity to gamma‐aminobutyric acid type A receptors (GABA_A_‐Rs) and potentiate the effects of GABA, are widely prescribed for anxiety, insomnia, epileptic discharge, and as anticonvulsants. The long‐term use of BZDs is limited due to adverse effects such as tolerance, dependence, withdrawal effects, and impairments in cognition and learning. Additionally, clinical reports have shown that chronic BZD treatment increases the risk of Alzheimer's disease. Unusual GABA_A_‐R subunit expression and GABA_A_‐R phosphorylation are induced by chronic BZD use. However, the gene expression and signaling pathways related to these effects are not completely understood. In this study, we performed a microarray analysis to investigate the mechanisms underlying the effect of chronic BZD administration on gene expression. Diazepam (DZP, a BZD) was chronically administered, and whole transcripts in the brain were analyzed. We found that the mRNA expression levels were significantly affected by chronic DZP administration and that lipocalin 2 (*Lcn2*) mRNA was the most upregulated gene in the cerebral cortex, hippocampus, and amygdala. Lcn2 is known as an iron homeostasis‐associated protein. Immunostained signals of Lcn2 were detected in neuron, astrocyte, microglia, and Lcn2 protein expression levels were consistently upregulated. This upregulation was observed without proinflammatory genes upregulation, and was attenuated by chronic treatment of deferoxamine mesylate (DFO), iron chelator. Our results suggest that chronic DZP administration regulates transcription and upregulates Lcn2 expression levels without an inflammatory response in the mouse brain. Furthermore, the DZP‐induced upregulation of Lcn2 expression was influenced by ambient iron.

**Abbreviations**
*Actb*: beta‐actin
Amg: amygdala
BZDs: benzodiazepines
CaMKII: calcium/calmodulin type II
*Crhbp*: corticotropin‐releasing hormone‐binding protein
Ctx: cortex
*Cxcl1*: chemokine (CXC motif) ligand 1
DFO: deferoxamine
DZP: diazepam
*Fabp7*: fatty acid‐binding protein 7
GABA_A_‐Rs: GABA_A_ receptors
GABA: *γ*‐aminobutyric acid
Hip: hippocampus
*Ifitm3*: interferon‐induced transmembrane 3
*Il‐6*: interleukin‐6
*Lcn2*: lipocalin 2
*Lyz2*: lysozyme 2
MAP: mitogen‐activated protein
Ngal: neutrophil gelatinase‐associated lipocalin
*Npy*: neuropeptide Y
*Ppia*: peptidylprolyl isomerase A
*Rps18*: ribosomal protein S18
*Selp*: selectin platelet
*Tbp*: TATA box‐binding protein
*Tnf‐α*: tumor necrosis factor‐*α*
*Vwf*: von Willebrand factor homolog

## Introduction

*γ*‐aminobutyric acid (GABA) is an inhibitory neurotransmitter that activates ionotropic (ligand‐gated ion channel) GABA type A receptors (GABA_A_‐Rs) in the mammalian brain. GABA_A_‐Rs are pentameric ion channels composed of seven subunit families (Sieghart 1995; Whiting et al. 1999). Most native GABA_A_‐Rs in the adult brain are composed of combinations of *α*,* β*, and either *γ* or *δ* subunits. GABA_A_‐Rs associate with higher brain functions, such as cognition, learning, and emotion (Collinson et al. 2002; Maubach 2003; Morris et al. 2006). The dysfunction of GABA_A_‐R‐mediated GABA systems has been implicated in neuropsychiatric diseases, including anxiety, depressive disorder, epilepsy, insomnia, and schizophrenia (Mohler 2006; Charych et al. 2009; Hines et al. 2012). Benzodiazepines (BZDs) are clinically used to treat anxiety, insomnia, and some forms of epilepsy and as adjunct treatments in both depressive disorder and schizophrenia (Bandelow et al. 2008; Rudolph and Knoflach, 2011; Volz et al. 2007). BZDs bind GABA_A_‐Rs at a high‐affinity binding site located between the *α* and *γ* subunits and potentiate GABA_A_‐R activities (Sigel and Buhr 1997). Although BZDs are frequently prescribed because of their high efficacy and low toxicity, there are significant risks associated with their long‐term use, such as tolerance, dependence, withdrawal, and cognitive and learning impairment (Golombok et al. 1988; Rummans et al. 1993; Zeng and Tietz 1999; Paterniti et al. 2002; Katsura et al. 2007; Vinkers et al. 2012). Furthermore, a recent clinical study revealed that BZD overuse is associated with an increased risk of Alzheimer's disease (Yaffe and Boustani, 2014; Imfeld et al., 2015; Rosenberg, 2015). The adverse effects following chronic BZD treatment are complex processes that remain incompletely understood.

Several studies have identified neuroadaptive mechanisms underlying BZD tolerance and withdrawal, including alterations in GABA_A_‐R subunit mRNA expression (Vinkers et al. 2012; Gutierrez et al., 2014; Wright et al. 2014). For example, repeated DZP (a medication in the BZD family) administration increased *α*1, *α*4, *β*1, and *γ*3 subunit mRNA levels and decreased *β*2 subunit mRNA levels (Holt et al., 1996). Additionally, the hybridization signals for N‐methyl‐D‐aspartate (NMDA) glutamate receptor mRNA were increased in the hippocampal dentate gyrus of rats administered BZDs (Perez et al., 2003). The *β* and *γ* subunits of GABA_A_‐Rs have phosphorylation sites in intracellular loops and these sites are phosphorylated by various kinases, such as protein kinase A (PKA), protein kinase C (PKC), tyrosine kinase Src, and calcium/calmodulin type II (CaMKII)‐dependent kinase. GABA_A_‐R phosphorylation could affect channel plasticity and surface trafficking mechanisms (Brandon et al., 2002; Kittler and Moss, 2003; Houston et al., 2007; Hu et al., 2008). These phosphorylation processes are likely associated with neuroadaptive mechanisms because DZP administration decreases CaMKII*α* and MAP kinase phosphatase mRNA levels in the mouse cerebral cortex (Huopaniemi et al., 2004). However, gene expression and signaling pathways relating to disadvantage of chronic BZDs treatment are not known completely.

In this study, the global transcription profiles of the mouse brain were investigated via microarray analyses to identify the genes and pathways that are associated with adverse effects following chronic DZP treatment. Epileptic effects are known to be frequently focused in the cerebral cortex and hippocampus. The amygdala is a neuronal substrate for emotional states such as fear and anxiety. Because both epileptic and anxiety disorders are commonly treated with DZP, mRNA expression levels in the cerebral cortex, hippocampus, and amygdala were the focus of the analysis in this study. The mRNA expression levels of some genes were significantly up‐ or downregulated by chronic DZP administration. Notably, we found that *Lcn2* mRNA expression was threefold higher in the DZP‐administered brains than vehicle‐administered brains. The analysis of microarray data and qRT‐PCR results revealed that the mRNA of the Lcn2‐001 splice variant (RefSeq# NM_008491, Ensembl# ENSMUST00000050785) was upregulated, consistent with Lcn2 protein expression levels. The immunohistochemical analysis revealed that Lcn2 protein was localized in neuron, astrocyte, and microglia. These results indicated that chronic DZP administration affected the transcription and upregulation of *Lcn2* expression and function in CNS, and that DZP‐induced Lcn2 upregulation was correlated with iron homeostasis.

## Materials and Methods

### Animals

Approximately, 8‐week‐old male C57BL/6 mice were used in our experiments. The mice were housed at 24 ± 2°C with a 12/12‐h light/dark cycle (lights on at 8:00 am) and were given free access to commercial food and tap water. The experimental procedures were based on the Guidelines of the Committee for Animal Care and Use of Hirosaki University, and all efforts were made to minimize the number of animals used and their suffering.

### Chemicals

DZP was purchased from Wako Pure Chemical Industries (Osaka, Japan). DZP was dissolved in intralipid (20% i.v. fat emulsion). Deferoxamine mesylate (DFO), iron chelator, was purchased from Abcam (ab120727; Cambridge, UK), and was dissolved in saline.

### Chronic treatment of DZP and DFO

To study the effect of chronic DZP treatment on gene expression, the mice were treated twice daily (at 8:00 a.m. and 5:00 p.m.) for 10 consecutive days with DZP (5 mg/kg i.p.) or Intralipid (Vlainic and Pericic, 2009; Wright et al. 2014). For the experiment of iron chelation, DFO (100 mg/kg i.p.) was administered with DZP or intralipid.

### RNA isolation

After the termination of repeated DZP or vehicle treatment, the mice were deeply anesthetized with a medetomidine‐midazolam‐butorphanol combination and were transcardially perfused with modified artificial cerebrospinal fluid (ACSF). The solution contained the following components (in mmol/L): 220 sucrose, 2.5 KCl, 1.25 NaH_2_PO_4_, 10.0 MgSO_4_, 0.5 CaCl_2_‐2H_2_O, 26.0 NaHCO_3_, and 30.0 glucose (330–340 mOsm). The brains were then quickly removed and cut into 1‐mm‐thick coronal slices with brain matrix. The cerebral cortex, hippocampus, and amygdala were punched out from the coronal slices and preserved in RNAlater (Ambion, Austin, TX) solution according to the manufacturer's instructions and were stored at −80°C for further processing. The total RNA was extracted from the punched out tissue of the control or chronic DZP‐administered mouse brains using an RNeasy Lipid Tissue Mini Kit (QIAGEN, Valencia, CA) in accordance with the manufacturer's protocol.

### Microarray analyses

Gene expression profiling was performed using a GeneChip^®^ Mouse Transcriptome Array (MTA) 1.0 (Affymetrix, Santa Clara, CA). The total RNA (100 ng) was assessed using first‐strand cDNA synthesis, second‐strand cDNA synthesis, and cRNA amplification. After reaction termination, the RNA concentration was measured using a NanoDrop spectrophotometer (NanoDrop Technologies, San Diego, CA, USA), and the size distribution (100 ~ 4500 nt) was checked via 1.5% denatured agarose gel electrophoresis. The cRNA (15 *μ*g) was subsequently synthesized with second‐cycle single‐strand cDNA. After template RNA removal, the single‐strand cDNA was purified. The cDNA (5.5 *μ*g) was then fragmented and labeled. cRNA and cDNA syntheses were performed using a GeneChip^®^ WT PLUS Reagent Kit (Affymetrix) in accordance with the manufacturer's protocol. Fragmented and labeled cDNA were injected and hybridized onto an MTA cartridge. After hybridization, the array cartridges were washed and stained. Fragmentation, labeling, and hybridization were performed using a GeneChip^®^ Hybridization, Wash, and Stain Kit and a GeneChip^®^ Fluidics Station 450 (Affymetrix). The MTA cartridges were scanned with a GeneChip^®^ Scanner 3000 7G System. After scanning the MTA cartridge, an array quality check (QC) was performed using Expression Console™ software (Affymetrix). We ascertained whether all array data fulfilled the following criteria: Pos_vs_neg_auc > 0.7, Poly (A) spike control: Dap‐5 >  thr‐5 >  phe‐5 >  lys‐5, and hybridization control: Cre‐5 >  bioD‐5 >  bioC‐5 >  bioB‐5 (the QC metrics recommended by the manufacturer). All array data from all samples analyzed in this study fulfilled those criteria.

### Whole‐transcriptome array

After the QC, the array data sets were analyzed via Transcriptome Analysis Console (TAC) software to identify genes. TAC software enables the easy investigation of expression levels of alternative splicing variants. Unpaired one‐way ANOVA was performed to compare the signal intensities indicating gene expression between the control and chronic DZP‐treated mice. Several genes that were significantly altered with *P*‐values <0.05 and fold changes ≥ 1.5 or ≤ 0.66 with chronic DZP treatment were selected and further analyzed. The array data are deposited in the Gene Expression Omnibus (GEO). The raw data can be viewed and analyzed (accession number GSE76700, http://www.ncbi.nlm.nih.gov/geo).

### Alternative splicing analysis

The expression levels from the microarray data of each exon were compared between the controls and those that received chronic DZP treatment. The values of signal intensities (log2) of particular genes and each probe selection region (PSR) were obtained using TAC software. The log2 values were converted into the antilog values to calculate the relative expression level of each PSR. The values of each PSR were divided by the signal intensity of the entire gene in question. To clarify which PSRs were altered by chronic DZP treatment, the value at baseline of the entire gene in the same region as the control was used.

### Gene ontology (GO) analysis

Differentially expressed genes were subjected to GO analysis using the PANTHER Classification System (http://pantherdb.org/), a part of the Gene Ontology Reference Genome Project. The gene sets that showed significantly different expression between the control and chronic DZP‐administered mice were analyzed, and the signal intensity ratio was illustrated using a pie chart of biological process and molecular function. The gene list input to the PANTHER Classification System was divided according to down‐ or upregulation by DZP regardless of the brain region.

### Quantitative real‐time reverse transcription PCR (qRT‐PCR)

The total RNA was extracted from mice as described above. Five microgram of RNA was reverse‐transcribed to cDNA using SuperScript™ III Reverse Transcriptase (Invitrogen, Carlsbad, CA) in accordance with the manufacturer's protocol. Real‐time PCR was performed using SsoFast™ EvaGreen^®^ Supermix (Bio‐Rad; Hercules, CA) in a CFX96 Real‐Time PCR Detection System (Bio‐Rad). The primer sets used in this study are shown in Table 1. The cDNA derived from transcripts that encode *Gapdh* was amplified in each sample as an internal control.

**Table 1:** The primer sets used in this study

Gene symbol	Official ID	Primer sequence (5′→3′)
*Lcn2*	ENSMUST00000192241 (PS‐A)	F: AAGGAAGCTGCACAGGGTCT
		R: GTGCAAGGTTGAGCAACAGG
	NM_008491 (PS‐B)	F: ATGTCACCTCCATCCTGGTCAG
		R: GCCACTTGCACATTGTAGCTCTG
	NM_008491 (PS‐C)	F: GGAACGTTTCACCCGCTTTG
		R: GAAGAGGCTCCAGATGCTCC
*Gapdh*	NM_008084NM_001289726	F: AGAACATCATCCCTGCATCCA
		R: CCGTTCAGCTCTGGGATGAC
*Ifitm3*	NM_025378	F: TTCTGCTGCCTGGGCTTCATAG
		R: ACCAAGGTGCTGATGTTCAGGC
*Selp*	NM_011347	F: AAGATGCCTGGCTACTGGACAC
		R: CAAGAGGCTGAACGCAGGTCAT
*Vwf*	NM_011708	F: AACAGACGATGGTGGACTCAGC
		R: CGATGGACTCACAGGAGCAAGT
*Lyz2*	NM_017372	F: TGCCAGAACTCTGAAAAGGAATGG
		R: CAGTGCTTTGGTCTCCACGGTT
*Fabp7*	NM_021272	F: CAGTCAGGAAGGTGGCAAAGTG
		R: GCTTGTCTCCATCCAACCGAAC
*Crhbp*	NM_198408	F: GTGGTCTTACCAGAAGGAGCATC
		R: AACCTTCCACTCGGAGTCTGAG
*NpY*	NM_023456	F: CGCTCTATCTCTGCTCGTGT
		R: TGTTCTGGGGGCGTTTTCTG

### Western blotting and densitometric analysis

The cerebral cortex, hippocampus, and amygdala were punched out from coronal brain slices of the control and the chronic DZP‐administered mice, homogenized, and sonicated in lysis buffer containing 20 mmol/L Tri‐HCl at pH 8.0, 0.32 mol/L sucrose, 1% TX‐100, 0.1% SDS, and cOmplete™ Protease Inhibitor Cocktail (Roche Diagnostics, Germany). Insoluble fractions were precipitated via centrifugation at 20,000*g* for 30 min, and the supernatant fractions were recovered as whole cell extracts. All steps in this procedure were performed on ice or at 4°C.

Equal amounts of protein were denatured in SDS sample buffer, separated on 5–20% gradient gels (Wako), and subsequently transferred onto a PVDF membrane (0.2 *μ*m, GE Healthcare Japan, Tokyo). The membranes were blocked with 5% skim milk in 0.1% Tween‐20‐TBS (T‐TBS, pH 7.6) at room temperature for 60 min. Then, the membranes were probed with primary antibodies at 4°C overnight. After washing the membranes with T‐TBS three times (10 min per wash), the membranes were probed with secondary antibody at 4°C for 2 h. Immunocomplexes were detected using ImmunoStar chemiluminescence reagent (ImmunoStar^®^ LD, Wako) and the ImageQuant LAS 4000 mini system (GE Healthcare). The detected band intensities were analyzed using ImageJ software (http://imagej.nih.gov/ij/). The following first antibodies were used in western blot analysis: anti‐Lcn2 (1:10,000, ab63929; Abcam) and anti‐Gapdh (1:5,000, FL‐335; Santa Cruz Biotechnology, Santa Cruz, CA, USA). Polyclonal goat anti‐rabbit IgG/horseradish peroxidase (HRP)‐conjugated secondary antibody (P0448) was purchased from DakoCytomation (Glostrup, Denmark).

### Immunohistochemistry

The mice were transcardially perfused with phosphate‐buffered saline, and the brains were removed and fixed with 4% paraformaldehyde for 48 h. After dehydration through a graded ethanol series, the blocks were embedded in paraffin, cut into 4‐*μ*m‐thick sections, and processed for double‐label immunofluorescence. Deparaffinized sections were incubated with a mixture of rabbit anti‐Lcn2 (1:500, ab63929, Abcam) and mouse anti‐NeuN (1:500, MAB377, Millipore, Bedford, USA), goat anti‐Iba1 (1:500, ab5076, Abcam), or goat anti‐GFAP antibodies (1:500, SC6170, Santa Cruz Biotechnology) overnight at 4°C. The sections were then rinsed and incubated with anti‐rabbit IgG tagged with Alexa Fluor 488 (1:1000, Invitrogen), anti‐mouse IgG tagged with Alexa Fluor 594 (1:1000, Invitrogen), or anti‐goat IgG tagged with Alexa Fluor 594 (1:1000, Invitrogen) for 2 h at 4°C. After rinsing, the sections were mounted with Vectashield (Vector Laboratories, Burlingame, USA) and observed with a fluorescence microscope (BZ‐ X700, Keyence, Japan). The numbers of Lcn2‐positive cells in the cerebral cortex, hippocampus, and amygdala were counted using ImageJ software (National Institutes of Health, Bethesda, USA) and presented as density. The area in which Lcn2‐positive cells were counted was also measured by ImageJ software.

### Statistics

Unless otherwise indicated, all numerical data are presented as the mean ± SEM. Statistical analyses of the gene expression levels using Affymetrix gene array data were assessed using one‐way ANOVA. All other comparisons in the analysis of alternative splicing, qRT‐PCR, and western blotting were assessed using Student's t‐test. Differences were considered statistically significant at *P *<* *0.05.

## Results

### Gene expression profile following chronic DZP administration

We used a DZP chronic administration mouse model followed by microarray analysis to understand the effects of chronic DZP treatment. Chronic DZP treatment altered the expression levels of a number of genes. Following chronic DZP treatment, there were 14, 64, and 13 downregulated genes and 43, 27, and 32 upregulated genes in the cortex, hippocampus, and amygdala, respectively (Table 2).

**Table 2:** Number of genes altered by chronic DZP treatment

	Downregulated	Upregulated
Cortex	14	43
Hippocampus	64	27
Amygdala	13	32

**Figure 1:**
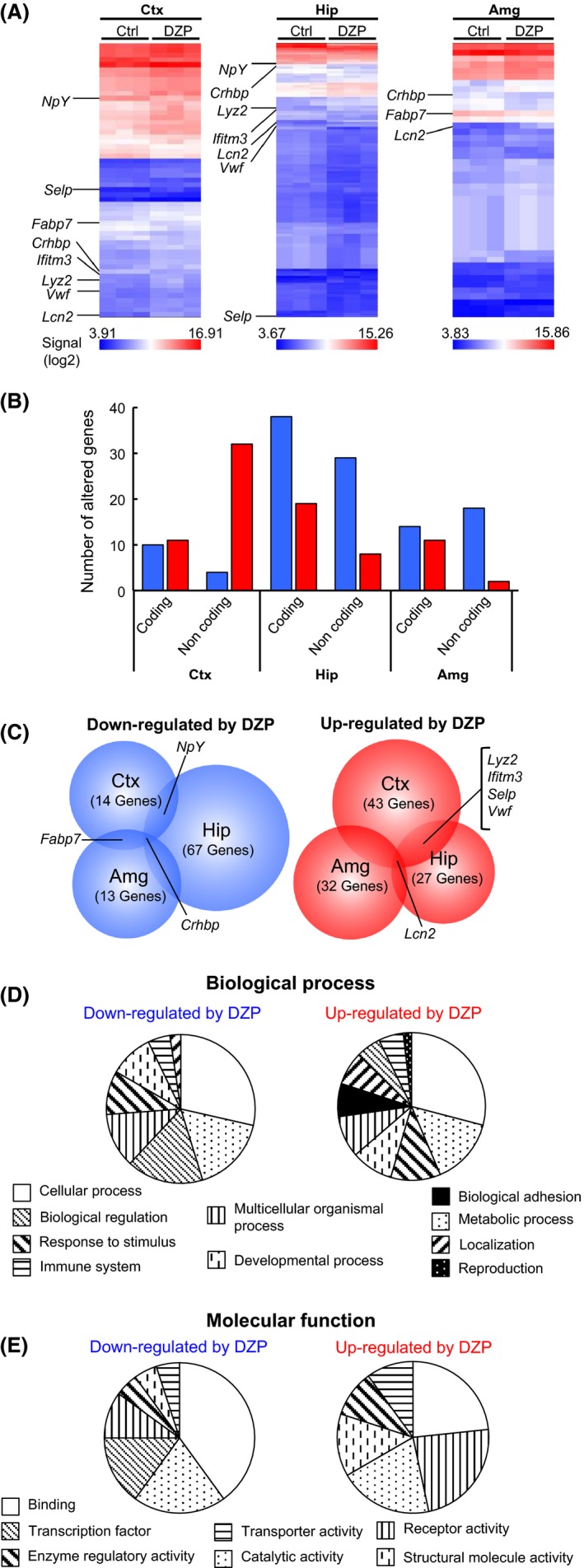
Global gene expression analyses of the cerebral cortex (Ctx), hippocampus (Hip), and amygdala (Amg) were performed via microarray in chronic DZP‐administered mice (DZP,* n* = 3) versus control mice (Ctrl, *n* = 3). In this analysis, the selection criteria are fold changes (1.5 ≤  or ≤ −1.5, *P *<* *0.05), except for pseudogenes and predicted genes. (A) Hierarchical clustering heat map of the mean signal intensity (log_2_) of the target gene and its scattering within the same group. Several genes that overlap in more than two regions of the brain are indicated. (B) The number of genes altered by DZP are shown in a graph that is divided into brain regions: coding or noncoding and down‐ (blue bar) or upregulated (red bar). (C) Venn diagram shows down‐ (left) or upregulated (right) genes following DZP treatment. The lists of altered genes were obtained through a GO analysis corresponding to (D) Biological process or (E) Molecular function via PANTHER with a pie chart. The list of genes was combined with all regions and divided as down‐ or upregulated following DZP treatment.

Significantly altered genes were determined according to criteria described in the Materials and Methods. Selected genes were summarized to a hierarchical clustering heat map (Fig. 1A) and are listed in Table S1. Furthermore, genes altered by DZP were classified as coding or noncoding (Fig. 1B). A total of 32 noncoding genes were upregulated in the cortex, and 67 genes (coding = 38, noncoding = 29) were downregulated in the hippocampus. These results suggest that chronic DZP treatment more strongly affected hippocampal gene expression compared with other brain regions.

To explore overlapping genes among brain regions, we generated a Venn diagram of genes that were altered by chronic DZP treatment in the mouse brain. The blue circle (Fig. 1C left) indicates downregulated genes. The *Npy* (neuropeptide Y) gene was downregulated in both the cortex and hippocampus, and the *Fabp7* (fatty acid‐binding protein 7) gene was downregulated in both the cortex and amygdala. The *Crhbp* (corticotropin‐releasing hormone‐binding protein) gene was downregulated in all three brain regions. The red circle (Fig. 1C right) indicates upregulated genes. *Lyz2* (lysozyme 2), *Ifitm3* (interferon‐induced transmembrane 3), *Selp* (selectin, platelet), and *Vwf* (von Willebrand factor homolog) genes were upregulated in both the cortex and the hippocampus. *Lcn2* expression was upregulated in all brain regions. Furthermore, we performed GO analysis using the PANTHER database to determine which types of alterations occurred within the cells via chronic DZP treatment. Both biological process (Fig. 1D) and molecular function (Fig. 1E) data were output from the GO analysis. For the output data, the biological processes and the molecular functions were categorized into groups. Altered genes were suggested to be involved in many biological processes, such as cellular process, metabolic process, and biological regulation (Fig. 1D). The expression levels of genes related to the molecular functions of binding, catalytic activity, and transporter activity were also altered. These results indicated remarkable relationship between chronic DZP administration and gene expression alterations of biological processes.

### Differentially expressed genes following DZP administration

**Figure 2:**
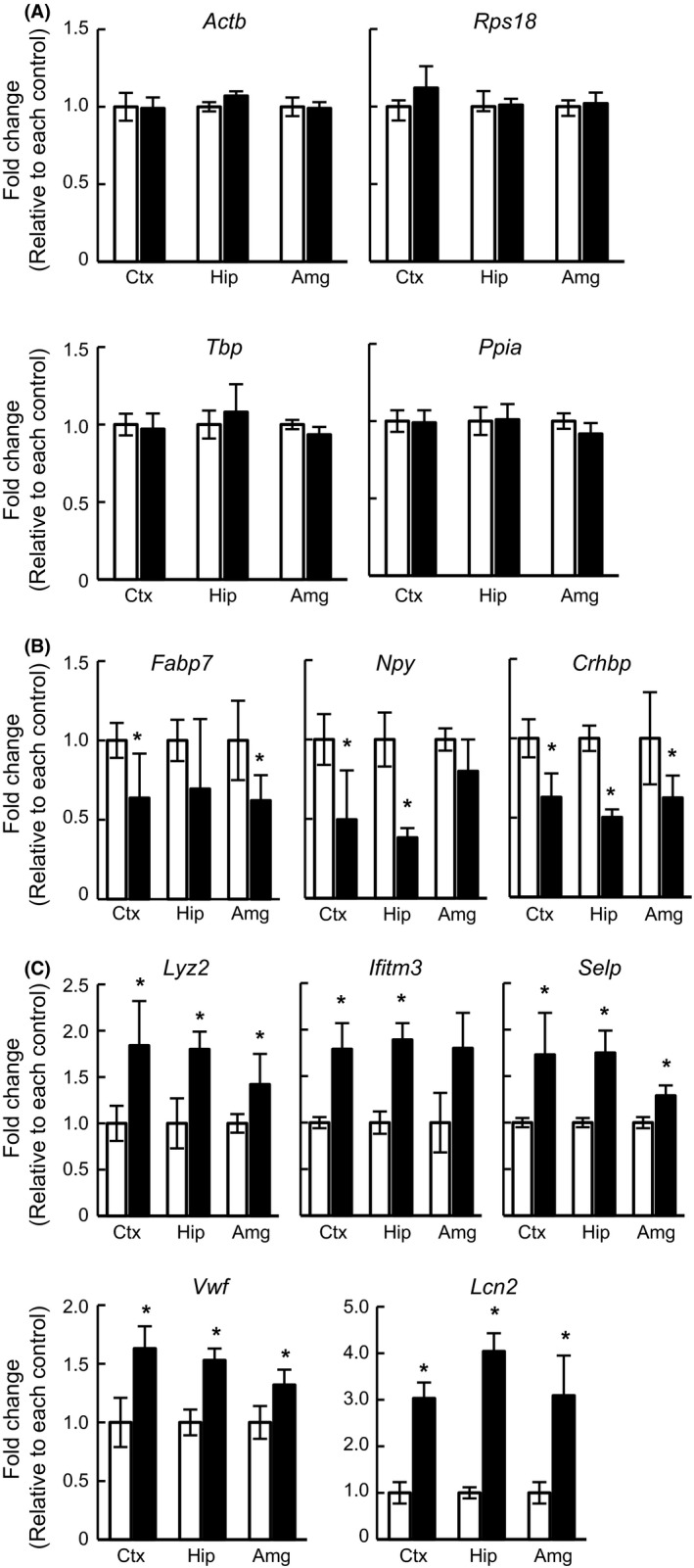
Gene expression levels of altered genes analyzed via microarray. The *Y*‐axis indicates the fold change compared with its control. Asterisks indicate statistically significant differences from the control (*: *P *<* *0.05, ANOVA). (A) The relative expression levels of housekeeping genes, such as *Actb*,* Rps18*,* Tbp*, and *Ppia*, were not altered following chronic DZP administration. (B, C) The relative expression levels of (B) down‐ or (C) upregulated genes following DZP treatment. White bar: control group, Black bar: DZP group.

**Figure 3:**
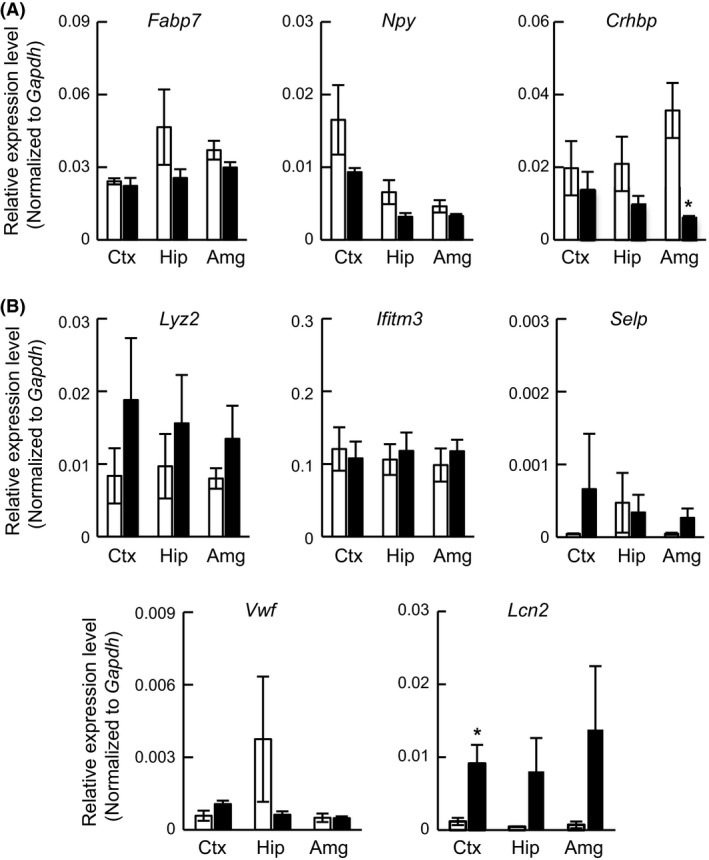
(A, B) Quantitative RT‐PCR analysis of (A) down‐ or (B) upregulated genes in microarray analysis. The expression levels were measured and normalized using the *Gapdh* (internal control) expression level. Data are expressed as the mean ± SEM,* n* = 3 per treatment group. Asterisks indicate statistically significant differences from the control (*: *P *<* *0.05). White bar: control, Black bar: DZP.

Next, we analyzed the expression levels of genes that were significantly altered in two or more regions according to the microarray data. The expression levels of 93 noncoding genes were altered by DZP; however, none of those expression profiles were altered in two or more regions. Therefore, noncoding RNAs were excluded from further analysis. Housekeeping genes, such as *Actb* (beta‐actin), *Rps18* (ribosomal protein S18), *Tbp* (TATA box‐binding protein), and *Ppia* (peptidylprolyl isomerase A), did not show significant differences (Fig. 2A). These results indicated that data from this microarray experiment were correctly obtained and reliable. The fold changes of downregulated genes (*Fabp7*,* Npy*, and *Crhbp*) are summarized in a bar graph (Fig. 2B, *P*‐value: see Table S1). *Npy* was significantly (0.38‐fold) decreased in the hippocampus but not in the amygdala. The fold changes of the upregulated genes, *Lyz2*,* Ifitm3*,* Selp*,* Vwf*, and *Lcn2,* are shown in a bar graph (Fig. 2C). There were significant differences in *Lyz2* and *Selp* gene expression in the amygdala (*P *=* *0.045 and 0.007, respectively); however, these differences did not fulfill our selection criteria for fold change (1.42‐ and 1.29‐fold, respectively). *Lcn2* underwent 3.03‐, 4.04‐, and 3.09‐fold increases, respectively, in each region compared with its control; in fact, the fold changes in *Lcn2* gene expression were the highest among all analyzed genes. These up‐ or downregulated mRNA expression levels by chronic DZP treatment were verified by qRT‐PCR (Fig. 3). Except for Lcn2, apparent changes were not observed by qRT‐PCR analysis. These results suggested that *Lcn2* gene expression was particularly affected by chronic DZP treatment.

### Alternative splicing analysis of Lcn2

*Lcn2*, also known as neutrophil gelatinase‐associated lipocalin (*Ngal*), *Siderocalin,* or *24p3,* has been reported to have six types of transcripts according to the Ensembl Genome Browser (http://www.ensembl.org/). Lcn2‐001 (NM_008491,ENSMUST00000050785) and Lcn2‐006 (ENSMUST00000192241) are protein‐coding splicing variants. Lcn2‐002 (ENSMUST00000136509), Lcn2‐004 (ENSMUST00000155830), and Lcn2‐005 (ENSMUST00000144569) are thought to contain intronic sequences and are not translated into proteins. Lcn2‐003 (ENSMUST147219) does not contain an open reading frame and is not translated to protein. However, it was unknown which splicing variant of *Lcn2* was regulated following chronic DZP administration.

**Figure 4:**
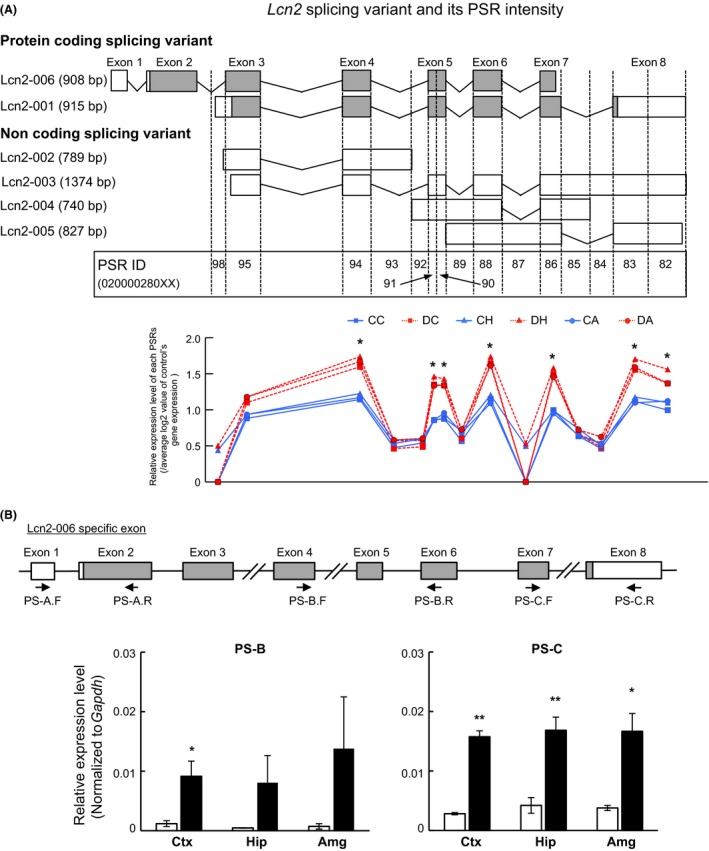
Alternative splicing variant analysis of the *Lcn2* gene. The gray and white boxes indicate translated and untranslated exon regions, respectively. The lines that connect each exon indicate intronic regions. (A) The schematic figure shows correspondence with *Lcn2* splicing variants (upper panel). Information about exons and PSRs was in accordance with a publicly available database, the Ensembl Genome Browser and NetAffx. The expression levels of each PSR are shown in a line graph (lower panel, blue line: control, red line: DZP). The *Y*‐axis indicates the relative signal intensities of PSRs against the entire *Lcn2* gene value in a control sample in the same region. (B) The results of qRT‐PCR of the *Lcn2* gene. The primer sets for *Lcn2* were designed against the indicated region (black arrow) and the expression levels of each region. The expression levels were measured and normalized using the *Gapdh* (internal control) expression level. Data are expressed as the mean ± SEM,* n* = 3 per treatment group. Asterisks indicate statistically significant differences from the control (*: *P *<* *0.05, **: *P *<* *0.01). White bar: control, Black bar: DZP.

This microarray system contains 162 probes that recognize introns, exons, and exon–exon junctions for *Lcn2*. These probes have been classified into 15 PSRs and five junction probe sets according to NetAffx (http://www.affymetrix.com/estore/index.jsp). Each PSR and junction contains at least four probes. To explore the relationship between the *Lcn2* splicing variant and PSRs, a schematic figure of the exons, introns, and PSRs of the *Lcn2* gene is shown in Figure 4A (upper panel). The probe intensities of all PSRs recognizing *Lcn2* exons were increased by DZP in every region. Furthermore, the probe intensity of PSRs that recognize introns, such as PSR02000093, 92, 89, 87, 85, and 84, were not altered by DZP (Fig. 4A lower graph). These results suggest that chronic DZP treatment upregulates Lcn2‐001 and 006 expressions in every brain region (Fig. 4B). Unfortunately, there were no PSRs to recognize Lcn2‐006 (exon 1: ENSMUSE00001336130 and exon 2: ENSMUSE00001337638) specifically in the Affymetrix microarray system. Therefore, the microarray data could not be used to elucidate which splicing variants of Lcn2 (Lcn2‐001 or 006) were upregulated. However, because the JUC0200014167 junction probes (which link PSR02000028086 to PSR02000028083) were highly expressed in the DZP sample (Table 3), we speculated that Lcn2‐001 expression was upregulated by chronic DZP treatment in mice.

**Table 3:** Average signal (log_2_) for the Lcn2 PSR and junction probe

Probe ID	Condition	Average signal (log_2_)
PSR0200028086	Ctx/Ctrl	8.68
	Ctx/DZP	13.04
	Hip/Ctrl	7.68
	Hip/DZP	12.26
	Amg/Ctrl	7.16
	Amg/DZP	10.98
JUC0200014167	Ctx/Ctrl	5.59
	Ctx/DZP	9.11
	Hip/Ctrl	4.74
	Hip/DZP	9.06
	Amg/Ctrl	4.07
	Amg/DZP	7.88
PSR0200028083	Ctx/Ctrl	9.74
	Ctx/DZP	13.57
	Hip/Ctrl	9.13
	Hip/DZP	13.21
	Amg/Ctrl	8.27
	Amg/DZP	11.96

To discriminate between Lcn2‐006 and other splicing variants, specific primer sets for exon 1 (forward) and exon 2 (reverse) were designed. Furthermore, we designed primer sets for ENSMUSE00001243567 (exon 4; forward) and ENSMUSE00001255227 (exon 6; reverse) to distinguish among Lcn2‐001, 003, 006, and Lcn2‐002, 004, 005. Additionally, specific primer sets were designed against ENSMUSE00000446298 (exon 7; forward) and ENSMUSE00000835504 (exon 8; reverse) to distinguish among Lcn2‐001, 005, and Lcn2‐002, 003, 004, 006. These primer sets were named PS‐A, PS‐B, PS‐C, and could measure the expression level of each splicing variant. Using these primer sets, the Lcn2 expression level in each exon was measured using qRT‐PCR. PS‐A was not detected in all samples (data not shown). As shown in Fig. 4B, PS‐B and PS‐C were increased in chronic DZP‐treated mouse brain samples. According to these results, chronic DZP treatment induced Lcn2‐001 expression in the cerebral cortex and hippocampus.

### Lcn2 expression at the protein level

**Figure 5:**
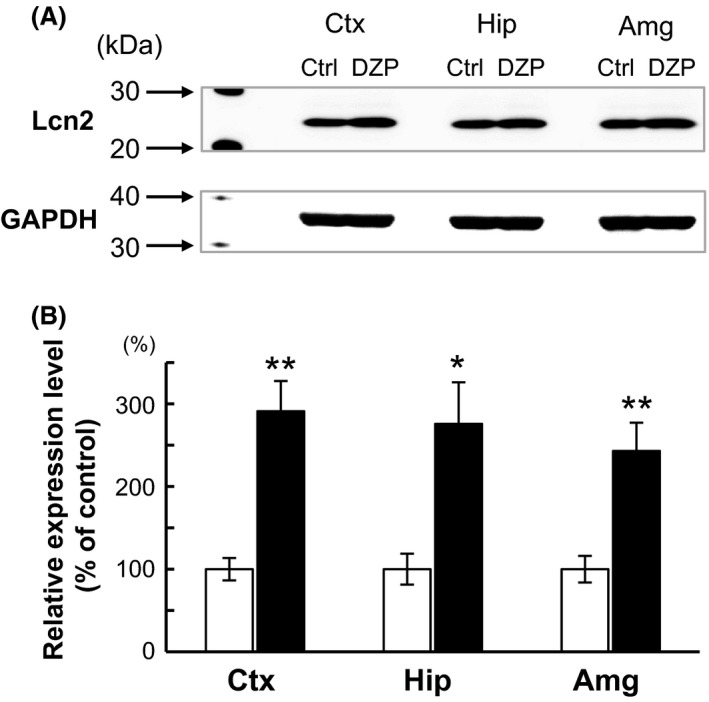
Lcn2 protein expression was increased following chronic DZP administration. (A) Western blot analysis of Lcn2 expression in the cortex (Ctx), hippocampus (Hip), and amygdala (Amg). (B) Western blot densitometric analysis. Lcn2 expression in the DZP group (black bar) was relatively high compared with the normalized control (Ctrl) group (white bar) in each region. Data are expressed as the mean ± SEM,* n* = 5 per treatment group. Asterisks indicate statistically significant differences from the control (*: *P *<* *0.05, **: *P *<* *0.01).

Next, Lcn2 induction by DZP was confirmed via western blot analysis. The Lcn2 protein of *Mus musculus* is composed of 200 amino acid residues, and the predicted molecular weight is 23 kDa. Its N‐terminal 20 amino acids are removed as a signal peptide, and the remaining 180 amino acids function as a mature protein. The molecular weight of this mature protein is approximately 21 kDa without posttranscriptional modification. As shown in Figure 5A, the Lcn2 protein was detected at approximately 21 kDa, as predicted above in all samples. Chronic DZP treatment significantly increased Lcn2 expression at the protein level (Fig. 5B). These results are consistent with the qRT‐PCR results (Figs. 3B, 4B).

### Immunohistochemical analysis for Lcn2‐positive cells

**Figure 6:**
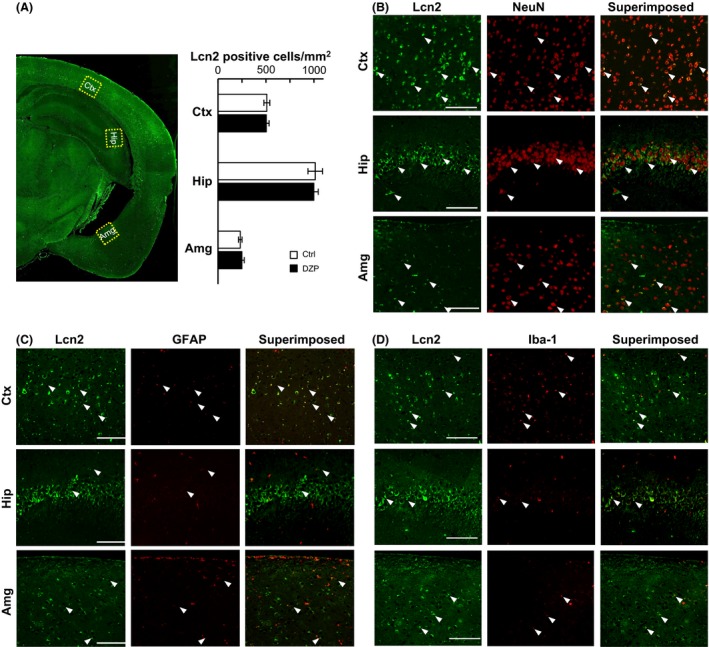
Immunohistochemical analysis to detect Lcn2 in the cerebral cortex (Ctx), hippocampus (Hip), and amygdala (Amg). (A) Photomicrograph showing the distribution of Lcn2 in a coronal section of control mouse. Lcn2‐positive cells in boxed area were counted and summarized in bar graph (Control: *n* = 4, DZP:* n* = 4). (B–D): Lcn2 was double stained with NeuN (B), GFAP (C), Iba‐1 (D). Each photomicrographs in B–D are enlarged view of the boxed area shown in (A). White arrowheads indicate some of double‐positive cells.

The expression level of Lcn2 was upregulated by chronic DZP treatment in cerebral cortex, hippocampus, and amygdala, however, Lcn2 expression was not unclear at the cellular level. To investigate whether Lcn2‐expressing cells were increased or not, and what type of cells accumulate Lcn2, immunohistological analysis were performed. The density of Lcn2‐positive cells in cerebral cortex, hippocampus, and amygdala were not significantly different between control (*n* = 4) and chronic DZP‐treated (*n* = 4) groups (Fig. 6A). This result indicates that upregulation of Lcn2 expression was not caused by increase in the number of Lcn2‐containing cells. Double stains for Lcn2 and NeuN (marker for neurons), GFAP (marker for astrocytes), or Iba‐1 (marker for microglia) were performed to identify which type of cells contained Lcn2. In cerebral cortex, hippocampus, and amygdala, double‐positive signals were detected in neuron, astrocyte, and microglia (Fig. 6B–D), indicating that Lcn2 were contained in those types of cells.

### The effect of iron chelator on DZP‐induced Lcn2 upregulation

**Figure 7:**
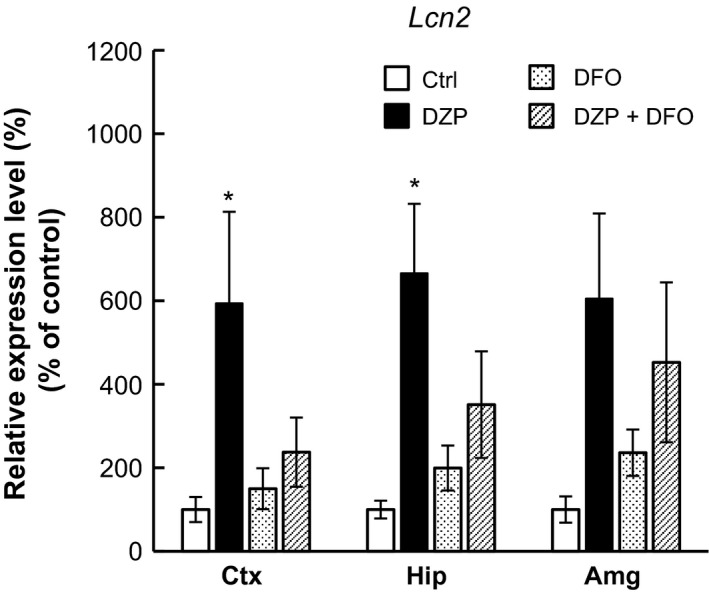
The effect of DFO treatment on *Lcn2* mRNA expression level was assessed by qRT‐PCR analysis. The Lcn2 expression levels of cerebral cortex (Ctx), hippocampus (Hip), and amygdala (Amg) were measured and normalized to expression level of each control (Ctrl) group. Data are expressed as the mean ± SEM,* n* = 3 per treatment group. Asterisks indicate statistically significant differences from the control (*: *P *<* *0.05). White bar: control, Black bar: DZP, dotted bar: DFO, Striped bar: DZP + DFO.

Some studies indicated that treatment of DFO reduced injury‐induced Lcn2 upregulation, and suggested the possibility that Lcn2 has function in iron homeostasis (Dong et al. 2013; Zhao et al. 2016). To examine the effect of DFO to DZP‐induced Lcn2 upregulation, qRT‐PCR analysis was performed using chronically DZP‐treated mice with or without DFO. The single chronic DFO treatment did not significantly affect to *Lcn2* mRNA expression. However, the significant or moderate upregulation of *Lcn2* mRNA expression by chronic DZP treatment was reduced by cotreatment of DFO (Fig. 7). This result suggested that DZP‐induced Lcn2 expression was regulated by iron in the CNS.

## Discussion

In this study, we performed global transcriptional analysis via microarray to investigate the effect of chronic DZP administration on the mouse brain and found significant alterations in the expression levels of 177 genes (Fig. 1 and Table 1). Affymetrix's gene chip probe for the mouse transcriptome assay detected not only protein‐coding genes but also nonprotein‐coding genes. Approximately, half of the significantly altered genes detected in the microarray system were nonprotein‐coding genes. In the cerebral cortex, 88.9% of the altered nonprotein‐coding genes were upregulated following DZP administration. Additionally, most of the nonprotein‐coding genes detected in the hippocampus and amygdala (78.4% and 90%, respectively) were downregulated (Fig. 1B). This result also implies that chronic DZP administration affects the expression levels of microRNA, pseudogenes, rRNA, and/or other noncoding RNA. Similarly, altered protein‐coding genes in each brain region exhibited individual differences according to the gene and the number of these genes (Fig. 1B, C). Furthermore, DZP had the greatest effect on mRNA expression in the hippocampus; this finding suggested that the mRNA expression levels could be regulated region‐specifically by chronic DZP treatment.

Lcn2 is a known member of the lipocalin family of transport proteins. Lcn2 is imported across the cell membrane during Lcn2 receptor (24p3R)‐mediated endocytosis (Devireddy et al. 2005; Yoon et al. 2006) and is secreted during exocytosis (Lee et al. 2012). At the cellular level of the central nervous system (CNS), Lcn2 is expressed in neurons (Naude et al. 2012; Xing et al. 2014), microglia, and astrocytes (Bi et al. 2013; Jang et al. 2013) and is released from those cells. The various functions of Lcn2 in the CNS have been reported. For example, Lcn2 released from injured neurons activates microglia and astrocytes (Xing et al. 2014). Activated microglia secrete Lcn2 and sensitize themselves to apoptosis (Lee et al. 2007). Lcn2 secreted from reactive astrocytes promotes neuronal death (Bi et al. 2013). In this study, we found the upregulation of Lcn2 expression following chronic DZP administration without increasing Lcn2‐positive cell number. In addition, Lcn2‐positive signal was detected in neuron, astrocyte, and microglia. However, because Lcn2 is secretable protein, it was not able to fully conclude that all cell types analyzed in this study express and secreted Lcn2. There is a possibility that glial cells express and secreted Lcn2, and subsequently Lcn2 was accumulated in neurons. At least, cell type‐specific localization of Lcn2 was not observed under the conditions of this study. Furthermore, it remains unknown how DZP regulates *Lcn2* mRNA expression levels. DZP is known as a positive allosteric modulator of GABA_A_‐Rs and enhances the inhibitory actions of GABA. GABA_A_‐R expression has been detected in not only neurons but also glial cells. Notably, a specific GABA_A_‐R‐mediated current response has been detected in astrocytes (Bormann and Kettenmann 1988; MacVicar et al. 1989; Steinhauser et al. 1994; Egawa et al. 2013) and cultured oligodendrocytes (Von Blankenfeld et al. 1991). This evidence suggests that chronic GABA_A_‐R modulation by DZP could occur in neurons, astrocytes, and oligodendrocytes. Additionally, Lcn2 expression levels may be directly or indirectly controlled by such GABA_A_‐R‐expressing cells. Lcn2 may be involved in the structural plasticity of neurons, such as reductions in spine density (Curto et al. 2016). Further analysis is needed to understand the relationship between Lcn2 expression and chronic GABA_A_‐R modulation.

The role of Lcn2 in DZP‐administered mice is unclear; Lcn2 upregulation in the brain after chronic DZP administration may affect iron homeostasis. Lcn2 is known to deliver siderophore‐bound iron to mammalian cells. Lcn2 binds to siderophore, and the complex is able to import via an Lcn2 receptor (Devireddy et al. 2005; Yoon et al. 2006). In some cell types, iron‐loaded Lcn2 increases intracellular iron levels, whereas iron‐lacking Lcn2 decreases intracellular iron levels (Devireddy et al. 2005; Richardson, 2005). In rat brain endothelial cells, the intracellular iron level was increased via Lcn2 application (Wu et al., 2015). Additionally, abnormal iron accumulation and the alteration of Lcn2 were reported in some neurodegenerative diseases, including Alzheimer's disease and multiple sclerosis (Berard et al. 2012; Naude et al. 2012; Weigel et al. 2014). Furthermore, astrocytic Lcn2 expression was triggered by biomolecules released from neuron, and neurotoxicity was induced via excessive iron delivery in Parkinson's disease model (Kim et al. 2016). This Lcn2‐induced neurotoxicity was increased by iron donor treatment, and was decreased by iron chelator treatment. On the other hand, DFO treatment reduced Lcn2 expression in brain injury model mice (Dong et al. 2013; Zhao et al. 2016). This evidence was consistent with our results of this study. Taken together, iron may have some role for the neurotoxic effect mediated by Lcn2 and the regulation of Lcn2 expression. The chronic modulation of GABA_A_‐Rs by DZP may be involved in the disruption of iron homeostasis due to the upregulation of Lcn2 expression without an inflammatory response, and the neurodegenerative effects (such as cognitive and learning impairment) could manifest as the adverse effects that are observed following chronic DZP administration (Golombok et al. 1988; Rummans et al. 1993).

Several studies have shown that Lcn2 expression is upregulated in various pathological conditions such as neurodegeneration, gliomas, brain injury, neuroinflammation, autoimmune disorders, encephalitis, hemorrhage, schizophrenia, and spinal cord injury (Reviewed by Jha et al., 2015). *Lcn2* gene expression was previously induced by several inflammatory stimuli, such as tumor necrosis factor‐*α* (TNF‐*α*), interleukin‐1*β* (IL‐1*β*), or interferon‐*γ* (IFN‐*γ*) or JAK2‐STAT5 pathway, in the 3T3‐L1 murine adipocyte cell line (Zhao and Stephens 2013). These signal transduction pathways have been intensively investigated in the immune system. Additionally, Lcn2‐related modulations of inflammatory responses in inflammatory or autoimmune disease conditions have been reported in Lcn2‐knockout mice (Berard et al. 2012; Shashidharamurthy et al. 2013). These factors indicate that chronic DZP treatment may be a potent inducer of proinflammatory genes in microglia. Regarding inflammation, cytokine expression levels were drastically increased in macrophages and microglia (Saglie et al. 1990; Banati et al. 1993). However, our microarray data showed that chronic DZP treatment did not affect the expression levels of proinflammatory genes such as *Tnf‐α*,* Il‐6*, or *Cxcl1*. The qRT‐PCR analysis also revealed that inflammatory‐related genes, such as *Tnf‐α*,* Il‐6*, and *Nos2* (*iNos*), were not altered (data not shown). Additionally, an immunohistochemical analysis revealed that signals of activated microglial markers (CD11b and CD68) were not detected in either control or chronic DZP‐treated mouse brain sections (data not shown). These results are in line with the fact that the expression levels of microglial proinflammatory genes were not increased following chronic BZD administration (Ramirez et al. 2016). These lines of evidence indicate that chronic DZP treatment did not lead to brain inflammation.

However, some of the genes altered by chronic DZP treatment were classified as relating to immune system processes in biological process by GO analysis (Fig. 1D). *Itpr1*,* Selp*, and *Cfh* (upregulated), along with *Nr4a3* and *Nptx2* (downregulated), were altered by chronic DZP treatment and were classified as immune system components. These genes are likely not induced by pro‐ or antiinflammatory responses. For example, *Nr4a3* is upregulated by Creb, a member of the leucine zipper family (Pei et al. 2006), in mice. The transcriptional activity of Creb is regulated by PKA or CaMKs (Brindle and Montminy 1992; Sassone‐Corsi 1995); thus, chronic DZP treatment is unlikely to be related to the inflammatory response. These items raise the possibility that the upregulation of *Lcn2* by chronic DZP treatment is not the result of an inflammatory response.

The alteration of DNA methylation is another hypothesis for the regulation of *Lcn2* expression. According to ENCODE at UCSC, there is a CpG island upstream of the *Lcn2* gene (chr2:32250995‐32252227 in NCBI37/mm9). However, this CpG island is present from the proximal promoter region to exon 1 of the *Ptges2* gene that is located next to the *Lcn2* gene; DNA methylation is not considered to be involved in *Lcn2* gene expression. Therefore, there is a possibility that another transcription factor(s) is activated by chronic DZP treatment and regulates *Lcn2* gene expression. Unfortunately, there are no significant peaks for various transcriptional factors in regulatory region of *Lcn2* in chromatin immunoprecipitation sequencing (ChIP‐seq) data from ENCODE at UCSC. Further investigation is necessary to clarify the signaling pathway of *Lcn2* gene expression following chronic DZP treatment.

In summary, we demonstrated that chronic DZP administration affected the expression levels of transcripts. This evidence suggests the possibility of additional effects of chronic DZP administration on signaling pathway and transcription modulation. Furthermore, the transcriptional and protein expression levels of Lcn2, which are related to iron transport, were upregulated without altering the expression levels of immunoreactive molecules. Chronic DZP administration may disrupt iron homeostasis, and cognitive and learning disorders could result.

## Authorship Contributions

T.F. developed the study concept. T.F., S.S., and Y.M. collected and analyzed the data. S.S. analyzed the Affymetrix microarray data. T.F. and S.S. wrote the first and final drafts of the manuscript. S.U. organized and directed the study. All other authors contributed to editing the manuscript.

## Disclosures

None declared.

## Supporting information


**Table S1.** The list of altered genes by chronic DZP treatment.
