# Supplementary material for: Chronic diazepam administration increases the expression of Lcn2 in the CNS
Source: Pharmacol Res Perspect. 2017 Jan 31;5(1):e00283. doi: 10.1002/prp2.283 (PMC5461642; doi:10.1002/prp2.283)
Supplement: Supplementary file 1 — Table S1. The list of altered genes by chronic DZP treatment. [file PRP2-5-e00283-s001.docx]

Supplemental Tabel S1: The list of altred genes by chronic DZP treatment.

| **Ctx** |  |  |  |
| --- | --- | --- | --- |
| **Public ID** | **Fold Change** | **ANOVA P-value** | **Gene Symbol** |
| NR_029543 | -2.11 | 0.005186 | Mir128-1 |
| NM_023456 | -2.02 | 0.011404 | Npy |
| NM_009215 | -1.97 | 0.00069 | Sst |
| NM_016789 | -1.88 | 0.047194 | Nptx2 |
| NM_011866 | -1.8 | 0.017716 | Pde10a |
| NM_010050 | -1.63 | 0.032316 | Dio2 |
| NR_030644 | -1.61 | 0.006209 | Mir582 |
| NM_198408 | -1.59 | 0.005933 | Crhbp |
| NM_021272 | -1.57 | 0.042328 | Fabp7 |
| ENSMUST00000103550 | -1.56 | 0.035635 | Ighv1-83 |
| XR_106184 | -1.55 | 0.016727 | D5Ertd683e |
| NR_045355 | -1.55 | 0.028591 | C130060C02Rik |
| NM_001043354 | -1.53 | 0.000323 | Rorb |
| NM_001134480 | -1.51 | 0.008257 | Plcxd2 |
| NM_008491 | 3.03 | 0.004394 | Lcn2 |
| NR_004414 | 2.88 | 0.024643 | Rnu2-10 |
| NR_028554 | 2.27 | 0.036942 | Snord70 |
| NR_028526 | 2.20 | 0.02196 | Snord49b |
| NR_046303 | 2.10 | 0.031767 | AF357426 |
| ENSMUST00000177688 | 2.01 | 0.043542 | n-R5s210 |
| NR_028552 | 2.00 | 0.016041 | Snord58b |
| NR_002851 | 1.95 | 0.006929 | Snord82 |
| ENSMUST00000083173 | 1.91 | 0.022993 | n-R5s88 |
| NM_017372 | 1.84 | 0.043956 | Lyz2 |
| NM_010591 | 1.83 | 0.037467 | Jun |
| NM_025378 | 1.79 | 0.011413 | Ifitm3 |
| ENSMUST00000093689 | 1.76 | 0.031463 | n-R5s211 |
| NR_028548 | 1.75 | 0.006726 | Snord16a |
| NR_028521 | 1.74 | 0.003197 | Snord11 |
| NM_011347 | 1.73 | 0.035664 | Selp |
| NR_037682 | 1.72 | 0.019353 | Snord42a |
| NR_028129 | 1.69 | 0.02285 | AF357399 |
| NR_028550 | 1.68 | 0.044354 | Snord49a |
| NR_028513 | 1.68 | 0.031738 | Snora73b |
| NM_011708 | 1.63 | 0.024129 | Vwf |
| ENSMUST00000083959 | 1.63 | 0.014419 | n-R5s151 |
| NR_028530 | 1.63 | 0.010178 | Snord66 |
| NR_002852 | 1.63 | 0.000496 | Rnu5g |
| NR_002852 | 1.63 | 0.000496 | Rnu5g |
| NM_001042605 | 1.62 | 0.001197 | Cd74 |
| NR_028542 | 1.61 | 0.04376 | Snord8 |
| NR_028547 | 1.60 | 0.03234 | Snord110 |
| NR_002903 | 1.60 | 0.012007 | Snord61 |
| NR_028531 | 1.59 | 0.017315 | Snord69 |
| ENSMUST00000093682 | 1.57 | 0.034062 | n-R5s58 |
| ENSMUST00000122599 | 1.57 | 0.016102 | n-R5s175 |
| NR_002842 | 1.57 | 0.000514 | Rnu3a |
| ENSMUST00000103407 | 1.57 | 0.000249 | Igkj3 |
| NR_034051; NR_106171 | 1.56 | 0.008624 | Snora34 |
| ENSMUST00000178208 | 1.53 | 0.01065 | n-R5s112 |
| ENSMUST00000180000 | 1.53 | 0.01065 | n-R5s120 |
| ENSMUST00000179377 | 1.53 | 0.01065 | n-R5s125 |
| NM_080455 | 1.52 | 0.017676 | Tshz2 |
| NM_001039562 | 1.52 | 0.013349 | Ankrd37 |
| ENSMUST00000082814 | 1.51 | 0.020006 | n-R5s28 |
| ENSMUST00000179195 | 1.51 | 0.004456 | n-R5s109 |
| NM_029137 | 1.51 | 0.000844 | Csmd2os |

| **Hip** |  |  |  |
| --- | --- | --- | --- |
| **Public ID** | **Fold Change** | **ANOVA P-value** | **Gene Symbol** |
| ENSMUST00000082403 | -5.66 | 0.000493 | mt-Ts1 |
| ENSMUST00000180349 | -4.3 | 0.000405 | LOC101056091 |
| ENSMUST00000082398 | -2.99 | 0.003966 | mt-Ta |
| NM_023456 | -2.62 | 0.000223 | Npy |
| NM_201530 | -2.59 | 0.004503 | Sly |
| uc029xyb.1; uc029xyd.1 | -2.59 | 0.004212 | 1700040F15Rik |
| ENSMUST00000178616 | -2.59 | 0.003294 | LOC101056096 |
| ENSMUST00000179336 | -2.4 | 0.002479 | LOC101055632 |
| uc029yfz.1 | -2.37 | 0.0035 | Orly |
| XR_106321 | -2.29 | 0.005632 | Srsy |
| XM_003689099 | -2.22 | 0.00161 | LOC100862006 |
| ENSMUST00000180837 | -2.07 | 0.029746 | 9330185C12Rik |
| ENSMUST00000083948 | -2.02 | 0.049946 | n-R5s168 |
| NM_001166758 | -2 | 0.010548 | Vmn1r159 |
| NM_198408 | -1.99 | 0.000069 | Crhbp |
| NM_001166837 | -1.93 | 0.017009 | Vmn1r114 |
| ENSMUST00000178024 | -1.9 | 0.03792 | G530012D18Rik |
| NM_001166750 | -1.89 | 0.005809 | Vmn1r143 |
| ENSMUST00000165025 | -1.86 | 0.01024 | Traj61 |
| NR_030447 | -1.86 | 0.039622 | Mir680-1 |
| NM_001166745 | -1.85 | 0.004212 | Vmn1r115 |
| uc007bte.1 | -1.84 | 0.013888 | Csprs |
| ENSMUST00000179529 | -1.83 | 0.014126 | Astx1a |
| ENSMUST00000178608 | -1.83 | 0.014126 | Astx1b |
| ENSMUST00000177724 | -1.83 | 0.014126 | Astx1c |
| ENSMUST00000177769 | -1.82 | 0.000985 | LOC101056018 |
| NM_001166744 | -1.82 | 0.005236 | Vmn1r116 |
| NM_001166848 | -1.79 | 0.01279 | Vmn1r130 |
| NM_001166849 | -1.79 | 0.01279 | Vmn1r137 |
| NM_001166747 | -1.77 | 0.001515 | Vmn1r135 |
| ENSMUST00000082423 | -1.77 | 0.010339 | mt-Tp |
| NM_001166740 | -1.77 | 0.012111 | Vmn1r125 |
| NM_001166752 | -1.77 | 0.04833 | Vmn1r152 |
| XM_003688870 | -1.77 | 0.002006 | LOC215866 |
| NM_001159743 | -1.75 | 0.015529 | Fam150b |
| NM_001166750 | -1.74 | 0.007922 | Vmn1r143 |
| NM_001166753 | -1.72 | 0.007511 | Vmn1r155 |
| NR_035489 | -1.66 | 0.011397 | Mir1963 |
| NR_106183 | -1.66 | 0.031519 | Mir8105 |
| NR_029568 | -1.65 | 0.047632 | Mir181a-2 |
| NM_001166749 | -1.65 | 0.010819 | Vmn1r142 |
| ENSMUST00000103605 | -1.65 | 0.048876 | Trav8d-2 |
| NM_001166738 | -1.64 | 0.037159 | Vmn1r104 |
| NM_001166726 | -1.64 | 0.024673 | Vmn1r127 |
| NM_001122661 | -1.63 | 0.000724 | Speer4e |
| ENSMUST00000179135 | -1.62 | 0.002432 | Astx2 |
| NM_001166723 | -1.59 | 0.00796 | Vmn1r94 |
| NM_001166743 | -1.59 | 0.019465 | Vmn1r117 |
| NM_001281511 | -1.58 | 0.000694 | Speer4c |
| NR_106087 | -1.57 | 0.036657 | Mir7228 |
| NM_001166714 | -1.57 | 0.019603 | Vmn1r122 |
| uc029vff.1 | -1.55 | 0.018565 | 4930578G10Rik |
| ENSMUST00000179723 | -1.54 | 0.021614 | Astx |
| NM_025759 | -1.54 | 0.004819 | Speer4d |
| XR_035373 | -1.53 | 0.016802 | AA474408 |
| NM_016789 | -1.52 | 0.00284 | Nptx2 |
| ENSMUST00000181084 | -1.52 | 0.001787 | C230088H06Rik |
| ENSMUST00000177683 | -1.52 | 0.016066 | Astx4a |
| ENSMUST00000179359 | -1.52 | 0.016066 | Astx4b |
| ENSMUST00000178390 | -1.52 | 0.016066 | Astx4c |
| ENSMUST00000123136 | -1.52 | 0.016066 | Astx4d |
| NR_045946 | -1.52 | 0.003812 | B020014A21Rik |
| NM_001166742 | -1.51 | 0.012038 | Vmn1r118 |
| XM_001473318 | -1.51 | 0.001128 | 1700066C05Rik |
| NM_008491 | 4.04 | 0.001408 | Lcn2 |
| NR_037241 | 2.16 | 0.044114 | Mir3080 |
| NR_046306 | 1.98 | 0.031845 | DQ267102 |
| NR_029563 | 1.93 | 0.004083 | Mir153 |
| NM_025378 | 1.89 | 0.002611 | Ifitm3 |
| NM_054037; NM_170727 | 1.82 | 0.048361 | Scgb3a1 |
| NM_017372 | 1.8 | 0.014244 | Lyz2 |
| NR_029762 | 1.8 | 0.007976 | Mir329 |
| NR_030575 | 1.77 | 0.039647 | Mir509 |
| NM_011347 | 1.75 | 0.007508 | Selp |
| NM_001271416 | 1.7 | 0.001948 | Ly6a |
| NM_013723 | 1.69 | 0.000742 | Podxl |
| NM_001146292 | 1.65 | 0.035334 | Celf4 |
| NM_001199351 | 1.65 | 0.027091 | Pnck |
| NM_007396 | 1.65 | 0.039508 | Acvr2a |
| NM_001276408 | 1.64 | 0.003293 | Fn1 |
| NM_001159518 | 1.64 | 0.027551 | Igfbp7 |
| NM_008597 | 1.62 | 0.015248 | Mgp |
| ENSMUST00000158355 | 1.6 | 0.02089 | Mir669m-2 |
| NR_028416 | 1.59 | 0.018435 | D630041G03Rik |
| NR_049186 | 1.57 | 0.027412 | Mir5615-1 |
| NM_009888 | 1.54 | 0.026895 | Cfh |
| NR_106158 | 1.54 | 0.024047 | Mir290b |
| NM_011708 | 1.53 | 0.002484 | Vwf |
| NM_027105 | 1.52 | 0.029978 | Krtap26-1 |
| NM_030018 | 1.51 | 0.020267 | Tmem50b |
| ENSMUST00000103374 | 1.51 | 0.028749 | Igkv1-35 |

| **Amg** |  |  |  |
| --- | --- | --- | --- |
| **Public ID** | **Fold Change** | **ANOVA P-value** | **Gene Symbol** |
| NM_015743 | -2.06 | 0.011057 | Nr4a3 |
| NM_001081134 | -1.93 | 0.030213 | Kcng1 |
| NM_153553 | -1.89 | 0.00151 | Npas4 |
| NM_177075 | -1.82 | 0.031697 | Fndc9 |
| NM_153155 | -1.81 | 0.049278 | C1ql3 |
| NR_029535 | -1.77 | 0.035149 | Mir99a |
| NM_031250 | -1.62 | 0.008435 | Ucn3 |
| NM_021272 | -1.61 | 0.00808 | Fabp7 |
| NM_183136 | -1.6 | 0.028882 | Spink8 |
| NM_198408 | -1.6 | 0.016615 | Crhbp |
| NR_106095 | -1.57 | 0.021018 | Mir7236 |
| NM_008795 | -1.54 | 0.031927 | Cdk18 |
| NM_027170 | -1.51 | 0.02511 | 2310057N15Rik |
| NM_008491 | 3.09 | 0.041668 | Lcn2 |
| NR_030601 | 2.2 | 0.014258 | Mir466d |
| NM_199058 | 2.15 | 0.041045 | Gpr6 |
| NR_106067 | 1.97 | 0.017552 | Mir7119; mmu-mir-7119 |
| NM_010076 | 1.9 | 0.034319 | Drd1a |
| ENSMUST00000103381 | 1.72 | 0.049397 | Ighm; Igkv8-27 |
| NM_146450 | 1.65 | 0.014722 | Olfr1314 |
| ENSMUST00000103461 | 1.64 | 0.026483 | Igh-VS107 |
| ENSMUST00000103898 | 1.63 | 0.033927 | Mir669o |
| NM_013616 | 1.63 | 0.007188 | Olfr65 |
| NR_030470 | 1.6 | 0.029365 | Mir669a-2 |
| NM_146903 | 1.6 | 0.00168 | Olfr871 |
| ENSMUST00000103883 | 1.59 | 0.000551 | Mir466b-8 |
| ENSMUST00000103910 | 1.59 | 0.000551 | Mir466e |
| NM_010585 | 1.59 | 0.046482 | Itpr1 |
| NM_147106 | 1.58 | 0.007116 | Olfr980 |
| OTTMUST00000117101 | 1.57 | 0.043755 | RP23-184F1.3 |
| NR_035408 | 1.55 | 0.018647 | Mir669a-1 |
| NR_037250 | 1.54 | 0.045018 | Mir669a-4 |
| NR_037251 | 1.54 | 0.045018 | Mir669a-5 |
| NR_037253 | 1.54 | 0.045018 | Mir669a-6 |
| NR_037255 | 1.54 | 0.045018 | Mir669a-7 |
| NR_037259 | 1.54 | 0.045018 | Mir669a-8 |
| NR_037260 | 1.54 | 0.045018 | Mir669a-9 |
| NR_037264 | 1.54 | 0.045018 | Mir669a-10 |
| NR_037266 | 1.54 | 0.045018 | Mir669a-11 |
| NR_037268 | 1.54 | 0.045018 | Mir669a-12 |
| NM_134241 | 1.54 | 0.015533 | Vmn1r212 |
| ENSMUST00000076614 | 1.54 | 0.033986 | Olfr1502 |
| NM_001039511 | 1.52 | 0.047952 | Ivns1abp |
| NM_001101488 | 1.51 | 0.01027 | Gsg1l |
| NR_029759 | 1.51 | 0.046333 | Mir325 |
